# Social richness, socio-technical tension and the virtual commissioning of NHS research

**DOI:** 10.1186/1478-4505-5-8

**Published:** 2007-06-06

**Authors:** Philip A Morgan, Christine A McCourt, Penny Youll

**Affiliations:** 1 Post-Doctoral Research Fellow, Centre for Research and Implementation of Clinical Practice, Faculty of Health and Human Sciences, Thames Valley University, 32-38 Uxbridge Road, Ealing, London, W5 2BS, UK; 2 Professor of Health and Social Sciences, Head of Post Graduate School, Thames Valley University, 32-38 Uxbridge Road, Ealing, London, W5 2BS, UK; 3 Senior Researcher, Evaluation and Development Unit, The Tavistock Institute, 30 Tabernacle Street, London, EC2A 4UE, UK

## Abstract

**Background:**

This paper draws on a recent study that evaluated the process of commissioning NHS funded research using virtual committees. Building on an earlier paper that reported our evaluation, here we focus on the effects of asynchronous computer mediated communication (CMC) when used to support group work.

**Methods:**

To do this the discussion focuses on how CMC affected three key group factors, building relationships, group cohesion and group commitment. The notion of socio-technical tension is elaborated and the paper explores how social richness can act to counter the socially impoverishing and time extending effects of asynchronous CMC.

**Results:**

We argue that social richness in this context results from the presence of five principal influences. These are: a dynamic range of participant aspirations and personal agendas; participant commitment to and identification with the work and ideals of the group; a rich diversity of social, professional and work-related backgrounds; a website designed to enhance participation and interaction and the mediating effects of an effective chairperson.

**Conclusion:**

If virtual work groups are to be used by the NHS in the future, then there is a need for more research into the role of social context and its relationship to the effectiveness of newly formed virtual groups. Equally as important are studies that examine the effects of socio-technical interaction on groups undertaking tasks in the real world of work.

## Background

The use of virtual groups in organisations is increasing and has created new possibilities for collaboration. The technology that makes this possible not only provides access to resources but also the ability to operate more flexible structures that bring geographically dispersed people and their knowledge and expertise together.

Much of the research concerned with computer mediated communication (CMC) and its application to virtual groups has been experimental [1,2], or has focussed on the evaluation of specific group support systems [3]. Most studies of the dynamics of virtual groups have been anecdotal and descriptive [4] or their applicability to 'real world' organisational settings has been 'jeopardised' because student subjects were used [5].

This paper draws on a recent study, undertaken by the authors that evaluated the commissioning of health research within the context of the NHS Health of Londoners Programme using virtual and face-to-face committees. The Health of Londoners Programme team hoped that using a bespoke website to enable commissioning group members to meet 'virtually' rather than face-to-face would widen access, and enable a clear, explicit process to underpin decisions that would lead to the production of relevant, well prioritised and high quality research. To facilitate evaluation they set up the Programme as a naturalistic experiment, with three theme groups allocated to use the website, over two nineteen-day meeting periods, and three groups allocated to the traditional model of two, three-hour face-to-face meetings. Our aim here is not to give a detailed account of the study and its findings; this was reported in an earlier issue of this journal [6] but rather to focus in more depth on a number of conceptual issues that were raised during the study about CMC. These include the effects of CMC when used to support group work and in particular how social richness can act to counter the socially impoverishing and time limiting effects of asynchronous computer mediated communication.

To do this we concentrate on the process as experienced by those taking part by exploring how participants related to the media and also how they interacted through the media. In particular we focus on how and in what ways the use of technology affected such key group factors as building relationships, group cohesion and group commitment. These are important because of the link between strong group member relationships and increased creativity and motivation [7] and improved performance [8]. We look at how a relatively diverse group of individuals can 'meet' in a 'thin' virtual environment and engage in a complex decision-making process. This is not automatically achieved, so we focus on how this occurs and what factors can support this. This paper seeks, therefore, to contribute to the wider debate around the use of virtual groups as well as to the specific application of this approach in the context of the real world of work within the NHS (UK).

### The context

At the inception of this study there was an established system for research commissioning at central and regional levels of the NHS. Regional offices, as well as taking a strategic management and planning role, commissioned research in priority areas relevant to their own region. Increasingly throughout the 1990's, the Department of Health and Regional Offices sought to involve a range of key stakeholders in setting the research agenda. Such stakeholders included both the providers and users of health services and reflected the influence of the 'user movement' and an increasing cultural climate of 'consumerism' both of which contributed to the creation, in 1998, of the Standing Group on Consumer Involvement in NHS Research [9].

During 2000, the London Regional Office set objectives for the Health of Londoners Programme which aimed to address issues known to affect the health of Londoners. These included high levels of deprivation, health inequality, mobility and ethnic diversity. A key part of this innovative programme was to create groups that included service users and practitioners, as well as researchers and those with health policy interests to carry out the commissioning of research in six priority areas.

To increase flexibility, to aid more inclusive participation and provide a more transparent process three of these groups formed 'virtual committees' using asynchronous, computer mediated communication and a bespoke meeting website. The remaining three maintained a traditional face-to-face committee approach.

In September 2001 funding for the programme was withdrawn. This was an effect of government restructuring of the NHS aimed at shifting the balance of power away from the centre to empower patients and staff. An immediate effect of this was the transfer of funds from DoH research to 'frontline services' [10]. Nevertheless, the decision was taken to continue the evaluation because of its anticipated value to future initiatives. At the time of withdrawal of funding, three committees including one virtual had completed both phases of commissioning. The other virtual committee had completed the first phase only (the phases of the programme are explained in 'The Commissioning Process' below).

### Literature relating to CMC

The literature highlights two principal perspectives taken to understand the media effects that influence social interactions and group dynamics online. The first of these is commonly known as the 'cues filtered out' hypothesis [11]. This hypothesis focuses on the reduction of non-verbal cues, and the less socially orientated and less personal nature of CMC, as the critical difference between face-to-face communication and CMC. The second perspective takes a relational stance [12] and is based on the premise that what happens in a virtual system is not the product of the technology alone but of social relationships, and other contextual factors, and the way these interface with the technology.

Perhaps the best known of the 'cues filtered out' group of theories are those concerned with media richness [13,14]. Media richness theory suggests that communication across various media differs depending upon the number of cue systems (bandwidth) within them [15]. The richness of a medium is defined in terms of how well it can communicate equivocal or ambiguous information. When messages are simple and unequivocal a lean medium, such as CMC, is considered sufficient. For more ambiguous, complex or emotional information, a richer medium, for example multi-media or face-to-face interaction, is generally seen as necessary.

Such perspectives on media effectiveness have been increasingly challenged, mainly because of their static nature. For example, Walther [15], in an attempt to understand what occurs over time in CMC groups, proposed the 'social information processing' (SIP) perspective. The basic assumption of this perspective is that communicators in CMC, like all communicators, are driven to develop social relations. In groups using CMC this can be problematic because users become acquainted with others through textually conveyed information that contains relatively less social information per message than in face-to-face groups.

This limited-capacity textual channel, Walther argues, tends to lead to retardation of relational development. Thus, the key difference between this process in CMC and in face-to-face communication is not the amount of information exchanged but the rate at which it is exchanged. SIP proposes that social interaction processes in CMC are very similar to face-to-face but are slower and that given sufficient time can be just as effective.

More recent literature, however [16,17], calls into question the assumption that the relational properties of CMC are determined by its technical (bandwidth) capacity alone. For example, in two studies investigating the development of group cohesion and process satisfaction, Burke *et al*., [2] found that the richness of the media did not seem as significant as anticipated in the development and work of on-line groups. Their conclusions suggest that contextual factors particularly individual and collective motivation, were at least as important.

As a result of such studies, perspectives are moving away from the central importance of media bandwidth (while still acknowledging the distinctive character and constraints of CMC) toward approaches that give more consideration to socio-technical interaction. These approaches stress the central role of social context and social influences such as group attraction, accountability, support, duty and power relations, as well as motivation, in the formation, development and effective working of groups using CMC [18].

These perspectives suggest that media bandwidth is, therefore, only part of the story, with a host of socially determined variables impacting on how, why, when and where such media are used. This creates the possibility that, irrespective of bandwidth, the dynamic effects of social context create a situation where media richness is less important to the effectiveness of the group than might be assumed.

Based on the findings from this study, we argue that the participants who use it and the social context in which it is used can influence CMC, in terms of its 'richness'. In this way, how media are used can be affected by a context of 'social richness' that acts to augment technical bandwidth capacity. It is to the effects of this socio-technical interaction on the relative 'richness' of the media, in the case of our study, that the discussion now turns.

## Methods

The study used a primarily naturalistic approach, but with aspects of both observation and experimental research design [19,20]. As noted earlier, the programme was, in effect, a 'naturalistic experiment' that provided an opportunity for comparison between innovative and traditional modes of research commissioning. However, the small scale and early stage of the work, the variability of the topics under consideration and the characteristics of the membership of the virtual committees meant that the study focused primarily on the experiences and perceptions of those taking part and observation of the process.

An action research approach was chosen to engage participants and programme staff in the study and to support learning and the creation of new knowledge through active collaboration [21]. Within this framework a number of methods were used. These included documentary analysis, semi structured interviews, observation, collaborative enquiry groups and questionnaires.

Documentary analysis of relevant policy and committee papers provided insight into the programme aims and objectives against which the process could be evaluated. Semi-structured, face-to-face interviews were conducted with a 50% purposive sample of participants across the five groups (32 interviews were conducted). This sampling approach was intended to ensure a mix of work roles, backgrounds and levels of prior experience of research commissioning.

Observation of each meeting provided descriptive data on the operation of the commissioning process in both modes. In the case of 'virtual' groups, the observation was by regular log-ins to the site, in the manner of 'virtual' participant observation [22]. A log-in diary was kept of observer impressions and all contributions to the meeting were downloaded as text files for subsequent analysis. Where meetings had already been conducted, it was still possible to download such files for analysis, giving a full transcript of contributions to the site. In comparison, minutes of face-to-face meetings provided only a brief record, with limited utility for analysis.

Three collaborative enquiry group meetings took place, one for each mode and a third as a combined discussion group. A total of 27 participants attended the three groups (6, 9 and 12 respectively). Written, telephoned or e-mailed comments were also received from 7 participants who were unable to attend. The collaborative enquiry groups generated wide-ranging discussion and valuable data that were used to verify and shape the themes identified in the main analysis.

The process of analysis combined the principles of grounded theory for the qualitative data [23] with more structured techniques [24] for data amenable to some quantification. For example, data such as levels and patterns of participation were mapped onto matrices to produce structured summaries. For interview and observation data, open coding of transcripts was used to identify emergent themes. On the basis of the findings and examination of the literature, social richness was identified as a core category for the subsequent selective framing and juxtaposition of relevant additional categories and codes. The themes and summaries were cross-checked and discussed within the research team to form the basis for discussion in the mode specific collaborative enquiry groups.

As mentioned above, the mode specific collaborative enquiry group discussions generated additional data as well as facilitating further analysis and validation of the themes. The themes were then mapped to a framework of outcome criteria developed from an earlier pilot study [25]. The outcomes of this mapping exercise formed the basis of a discussion paper circulated to participants prior to the final, combined, collaborative enquiry meeting where participants from both modes were able to discuss the themes further and compare their experiences directly.

## Results

### The participants

A key innovation of this programme was the widening of participation by advertising for membership of the commissioning committees. Advertisements were placed in the London press as well as national broadsheet newspapers and via practice and research based networks. Criteria for selection emphasised awareness of issues relevant to public health and the responsibilities of research commissioning groups. Specific criteria were stated as:

• Computer literacy

• Understanding the role of research in the NHS

• Familiarity with issues that affect London's health

The London Regional Office carried out selection and successful applicants were allocated to a committee based on their stated theme preference. As the committees would subsequently be randomly allocated to virtual or face-to-face modes, members were not aware in advance of the virtual 'experiment' or in which mode they would participate.

Preparation and training of committee members involved three key activities: orientation to the programme; orientation to the commissioning process and a research awareness workshop. During this face-to-face preparation members were introduced to their respective group and for the first time informed of the mode (virtual or face-to-face) in which they would be participating.

### Participant backgrounds

Most participants had a combination of backgrounds. For example, those who identified themselves primarily as practitioners also tended to have research experience or interests. Many academics/researchers also had clinical practice roles and a number who listed themselves as 'retired' had prior experience as practitioners, managers and researchers and were now active in a user or voluntary sector role. The number of consumers recruited was small and these members tended to have some research or health service working experience as well. It was noted that, in health, everyone is a sometime or potential user. The term 'consumer' here refers to someone whose main experience or interest is as a service user rather than as a professional or researcher who may also use the health services. Participation by role according to the self-identification of committee members is given in Table 1. Although all had experience of using computers, e-mail and the Internet, levels of confidence and the extent to which the medium played a part in their everyday lives, varied widely.

**Table 1:** Primary background of committee members.

**Committee**	**Consumer**	**Practitione**r	**Academic/Research**	**Policy/Manager**	**Vol. sector**	**Other**
Health service models (Virtual)	2	-	3	5	-	1 (a)
Regeneration (Virtual)	-	1	4	7	1	
Citizens & Consumers	2	1	6	3	1	1
Ethnicity	1	5	8			1 (b)
Older people & children	2	1	9 (c)	2		

(a) One member who described herself as a carer was also a health service manager. (b) Consumer and retired academic (c) Several now retired.

### The commissioning process

The overall commissioning process was conducted in two phases. In phase 1, committees established priority topics for research within their theme area. Phase 2 was concerned with reviewing proposals and selecting those projects to be commissioned. Committee members did not participate in the preparation of research briefs nor did they play a part in the ongoing monitoring of the commissioned research. These committees, both virtual and face-to-face, were to have a limited life, meeting solely for this round of commissioning and then disbanding.

The virtual 'meetings' mirrored face-to-face meetings in terms of process but differed in the time period allocated for each phase. Each phase of a virtual committee extended over a nineteen-day period as compared to a three-hour face-to-face meeting (see Table 2 for the sequence of activities). Programme officers from the regional office were 'present' during both phases and facilitated the process by responding to procedural questions and providing requested information. Each meeting also had a chairperson, appointed from within the membership by the Programme team.

**Table 2:** Virtual meeting sequence of activities.

**Days 1–2**	Introduction. Potential topic areas put forward, maximum one per member
**Days 3–9**	Discussion phase: exploration of potential topic areas
**Day 10**	Chair prepares summaries
**Days 11–14**	Voting. Members vote for their preferred three topic areas
**Day 15–16**	Vignettes prepared by project team
**Days 17–19**	Members view vignettes and make comments

**Phase 1.**	**Proposing, discussing and deciding on topic areas.**

**Pre day 1**	Members receive hard copies of proposals relevant to their sub-committee
**Days 1–7**	Discussion at sub-committee level
**Day 8**	Sub-committee Chairs prepare brief statements for General Forum. Remaining proposals needed for General Forum couriered to members.
**Days 9–15**	Discussion at General Forum level.
**Days 16–17**	Chair prepares summary of discussion and options portfolio
**Days 18–19**	Members vote on options

**Phase 2.**	Reviewing and selecting proposals for funding from within selected topic areas

### The virtual process

Four key factors emerged that had a bearing on the social context within which the virtual committees worked. First, these were zero-history, time limited groups treading a new path through the complexity of research commissioning on-line. Second, this was not an abstract experiment but 'real world' work carrying with it real responsibilities and consequences as well as possibilities. Third, the participants all had previous experience of committee work (to varying degrees) and of decision-making in general (not necessarily within the health services) and for the most part had professional backgrounds.

Finally, participants 'self-selected' to take part in this programme by responding to invitations placed as advertisements in a number of publications, both public and professional. Such self-selection foregrounds a variety of possible personal motivating factors, agendas and aspirations that were instrumental in prompting individuals to seek inclusion. It is against these contextual factors that the media effects found to be present during the process of virtual commissioning are discussed. To do this we focus on three closely interrelated key elements of group development: building relationships; group cohesion; and group commitment [32].

### Building relationships

Building team relationships is important to the effective working of virtual groups on a number of levels. Key to the success of virtual groups is effective communication which, in turn, depends on the personal relationships of group members [27]. Strong relational links have been associated with a number of group effectiveness factors which include; high task performance [8]; enhanced communication and more effective information exchange [28]; and enhanced creativity, motivation, increased morale and a more effective decision-making process [7]. In this study, in spite of the time-limited constraints of the commissioning programme, we found a number of examples of relationship building in the virtual committees.

At the very start of the programme, during the initial face-to-face preparation, participants were introduced to their respective groups. This provided an opportunity to begin to build group relationships and to set group norms. However, many found this opportunity for relational interaction difficult mainly because the chairperson of each committee was still to be appointed with the result that this key initial meeting lacked facilitation and focus as the following example illustrates:

The orientation meeting was good. People from a range of backgrounds were there. We got a lot of information. We were briefed on roles and so on, but we did not meet the chair of our group and go over how we would be working. We really didn't start to gel as a group (manager member).

This proved important because these committees were zero-history and time-limited groups. In such a situation there would be little subsequent opportunity to discuss the setting of ground rules and ways of working as the first step toward the development of group norms and shared understandings. These activities take time in face-to-face situations and even longer in virtual contexts [29].

Studies into how members of groups develop shared understandings and how this affects performance suggest that members of new groups base their actions, and interpret other member's actions, on the norms they hold as members of groups in similar situations [30]. The issue here is that the participants in the virtual committees had not been in such a situation before. Because of this, the norms they tended to apply reflected their experience of face-to-face group interaction: a situation that could in a virtual context result in uncertainty and anxiety [31] as is suggested here:

There wasn't the usual level of discussion you would expect in a face-to-face group. I felt very uncertain at times. I just had to wait and see what comments would be posted (academic/researcher, member).

As a result of growing anxiety among participants at not having had the opportunity to begin relational interaction in the initial face-to-face meeting, the chairperson of one virtual committee organised an additional face-to-face get-together. This took place between the two phases of the commissioning process. The purpose of the meeting was to bring the committee members together in a social setting in an attempt to 'make good' the relational deficit imposed by the virtual medium through which they were communicating. What is of interest here is the belief that face-to-face meetings are the most effective way of building group relationships and the apparent need to apply the relationship building processes of face-to-face meetings to the virtual context.

This brings us back to the work of Bettenhausen and Murnighan [30] and the suggestion that until participants reach an optimum level of individual and relational competence within the medium they will seek stability through familiar face-to-face experiences. Nevertheless, the anxieties and the active measures adopted to allay them reported in this study are, we suggest, indicative of a desire and determination on the part of the participants to build a relationally strong and socially rich environment.

### Group cohesion

Closely linked to group relationships is cohesion. Cohesion is defined as the aggregate of the interpersonal attractions of the individual group members to each other and to the group as a whole [32]. The importance of the notion of a cohesive group is linked to improved performance, increased satisfaction and lower group member turnover [26]. Cohesion is thought to increase when participants in a group perceive that individual and common goals can be achieved through group action [2]. Key to the development of cohesion are trust and interpersonal attraction.

The notion of swift trust put forward by Jarvenpaa et al [33] and later elaborated by Ishaya and McCaulay [34], seeks to explain how individuals who are 'thrown together' quickly become productive in spite of not developing solid interpersonal relations. Ishaya and McCaulay [34] report a link between trust and performance. The greater the trust the better the performance of the group and that developing and maintaining trust is contingent on a range of actions that contribute to the cohesion of the group.

Interpersonal attraction is, arguably, dependent on effective communication [32]. A particular challenge to effective communication in this study, and by definition to group cohesion, was the absence of visual and verbal cues. This had a number of effects. For example, the language of contributions in this textual medium was relatively formal and made up of composed statements with a tendency on the part of some participants to use the impersonal third person. The following extract from a discussion transcript, which represents a response to a previous contribution, provides an instance of this:

The proposal is not suggesting that NHS staffing should 'mirror' the surrounding population rigidly, only that there is potential to foster local regeneration through employment/training policies that provide employment opportunities for the local population, and also, very importantly, foster culturally sensitive care (manager, member).

Such restrained and semi-formal interaction supports the premise that these virtual committees were in the early stages of interpersonal and group development. Some participants, even at this early stage, were more successful, and skilled, than others in adopting a conversational style and responding to contributions directly, using examples, narratives or anecdotes from personal experience to illustrate their points, for example:

I am still trying to get my brains around the comments from the peer reviewers, but they were not convinced about this study, so I think Jane (name changed) has raised a very relevant point which is helpful at the start of the discussion (chairperson).

There are a number of studies that show CMC participants can, and do, use more socially orientated, richer, communication styles as they develop individual and relational competence but that, for most, this takes time [15,22,29,35].

The absence of social context cues affected communication and cohesion in other ways. Some participants reported an increased sense of individuality and, to some extent, a reduced sense of being a member of a group, for example:

I felt on my own at times. Lack of feedback meant I couldn't reflect on what others were saying. I felt very unsure because of that because I didn't know why they were not responding (manager, member).

The basis for this was mainly to do with issues of interactivity, such as the slow pace of discussion, delay in receiving feedback, and absence of visual cues that would normally aid interpretation of lack of response and the need for caution in the wording of textual messages. The latter reflected concerns about the visibility and finality of remarks that were effectively 'in print'. As a result of these reduced social cues effects, a number of participants described feeling alone, isolated or even threatened as this example suggests:

We didn't really know each other, it was a meeting of strangers really, I felt a bit uncertain, a bit vulnerable (consumer, member).

These responses can be interpreted from two perspectives. The first reflects the now conventional bandwidth related reduced social cues conception of CMC interaction. This interpretation acknowledges the complexity of managing, processing and producing textual messages with the added difficulty of a slower rate of information exchange that is a feature of CMC [15]. The sluggish rate of exchange can result in a state of 'environmental uncertainty' [36] that can prompt a textual communication style that is characteristically formal and impersonal [1]. Such a style is intended to communicate clearly in what is perceived to be a lean medium, open to misinterpretation [37]. The cumulative effect of the slow rate of interaction for some participants in this study was to create the disturbing impression of a virtual void. An impression that prompted one participant to enquire 'is anyone out there?'

The second perspective attempts to move away from the monolithic view of reduced social cues. The work of Watt *et al*., [18] argues that, whereas CMC may indeed filter out many interpersonal cues that identify and individuate the communicators, group level cues are often defined by the context in which the interaction takes place. As such group level cues can be communicated relatively independently of bandwidth and can have considerable influence over the interactions that occur.

Examples of group level cues in the context of this study include the original information participants received from the programme office that set out the aims and purposes of the programme. The process of self-selection, the subsequent allocation to groups and the meeting web site that contained group member biographies were rich in meaning. Such group level cues, according to Watt *et al *[18] can exert considerable influence over the self-perceptions and behaviours of those receiving this type of communication.

To follow this logic a step further, the group level cues perspective would argue that the concerns expressed by participants, and noted above, are examples of difficulties at the level of interpersonal cues, mainly as a result of bandwidth problems. At the level of group cues, participants perceived themselves to be members of a group and felt frustrated and anxious at being constrained, because of bandwidth effects, from functioning as they understood group members should, based on norms derived from their face-to-face experiences. This perspective would suggest that participants were indeed beginning to identify with their respective groups and that the tensions being played out represent a step towards group cohesion:

And it was only really towards the end of the second web based meeting that I felt we were beginning to gel into a team. (manager, chairperson)

### Group commitment

Whereas cohesion is essentially a property of the group as a whole, commitment reflects an individual's feelings of identification with the group's goals and tasks [26]. Here we discuss how a virtual meeting approach influenced commitment and motivation, how levels of commitment revealed themselves and the importance of the role of the chairperson in developing and maintaining motivation and commitment.

From the very beginning there was obvious commitment to the ideals and principles underpinning the programme. Participants expressed eagerness to take part because of the appeal of widening participation in research commissioning in particular and NHS decision making in general and also because of their interest to the area of focus of their commissioning group. The following provide an example of the range of motivations to be part of this programme:

Now I am retired I was interested in doing something of this kind, getting involved in public service on a voluntary basis (consumer/retired academic, member).

This country gave my parents somewhere to live, it gave me an education, I want to put something back (consumer, member).

I think it is beneficial for me as a researcher, to actually see the research process from both sides (researcher, member).

I think it is a very important and significant development. Users can give a very important perspective on what we want to do. It is public money we a re spending, it is good to widen it out from the usual suspects (academic/researcher/practitioner, member).

That participants self-selected for a place on the programme provides an indication of the potential level of commitment that existed. Indeed, when participants were informed of the withdrawal of funding there was dismay but also a tangible, and clearly expressed, determination to continue with the evaluation.

The level and pattern of participation can give a clue to the commitment of participants in a virtual committee context. Levels of participation were somewhat higher in phase 1 than in phase 2 in the only virtual committee to proceed through both phases (see Table 3). The level of discussion across both virtual groups in the first phase was extensive and described as 'lively'. The level of both discussion and general on-line interaction was reduced and became increasingly sparse as phase 2 proceeded.

**Table 3:** Levels of participation in the virtual groups

**Theme group (n. members)**	**Topics proposed (n)**	**Contributed to the discussion (n)**
Health service models (n 10)	7 topics proposed	Phase 1 = 6 Phase 2 = Nil (funding withdrawn)
Regeneration (n 13)	9 topics proposed	Phase 1 = 9 Phase 2 = 8

Patterns of interaction provide a valuable picture of individual commitment. A minority of participants made a number of contributions spaced throughout both phases. Interestingly it was these participants who also tended to be best at responding and interacting with others. Some made all or most of their contributions on a single day, which tended to have a negative effect on interaction across the group. Such patterns reflect the variability of individual commitment not uncommon in virtual contexts [38]. For Fahy *et al*., [38] commitment reflects personal investment in the work of the group. Committed participants actually interact; they do not simply log-on, contribute and leave, oblivious of others.

Clearly, motivation is a factor here and individual aspirations and personal agendas are important influences. However, in addition to these personal motivations, the findings from this study suggest that, in a virtual context, two key closely linked elements are necessary for individual and group motivation and commitment. These are: a rich, interactive virtual environment and an effective chairperson or facilitator.

While patterns of participation and levels of interaction in the virtual mode were reduced in phase 2 compared to phase 1 there was also a general feeling of a lack of momentum in phase 2. Some participants felt this was related to the extended time-scale within which the virtual committees operated. This, to an extent, is counter intuitive, because on the one hand the extended time provided more opportunities to contribute and on the other hand, there is a considerable literature to support the notion that CMC groups need longer to achieve consensus than face-to-face groups because of the effects of restricted bandwidth [1,39]. Indeed, Walther [12] found that compared to face-to-face groups, it takes four to five times longer for CMC groups to reach consensus when time was not restricted.

This highlights an important point. The studies into the effects of reduced social cues referred to above were based on experimental methodologies in non-real world situations using (mostly) student subjects. This raises the possibility that given a real world situation, such as in this study, a number of variables that make up and enrich the social context can interact with the media to foreshorten the time required to complete the task. As such, it may be that participant concerns about a lack of momentum have more to do with what might be described as a socio-technical tension. Such a tension appears to operate between, on the one hand, a rich and dynamic social context made up of motivated and experienced committee members and, on the other, the constraints imposed on time and social interaction by narrow bandwidth asynchronous CMC.

A socially rich virtual environment is a concept central to this discussion. Creating such an environment, according to Knoll and Javenpaa [31], is dependent on individual commitment and frequent proactive rather than reactive communication. Gunawardena and Zittle [40] capture this by pointing out that creating a rich environment is a multi-factorial endeavour. Important factors include choice of media, site design, social relationships, participation patterns, levels of involvement and the role of the chairperson or facilitator. The important point that comes through here is that the creation of a dynamic and rich virtual environment is not solely a technical problem. It is equally and perhaps more, to do with social and relational processes that are enabled by a range of factors that include appropriate media conditions such as a well designed site that enhances interaction.

The generally high levels of chairperson activity in this study provide an indication of the importance of their mediating role at the point of socio-technical tension. We found that they played a key part in ensuring continuity, encouraging participation, summarising points, explaining complexities, drawing links between contributions, inviting response and consolidating information. At times they also used the telephone and conventional e-mail to encourage participation.

The significance of this kind of activity is that it highlights the central place of the virtual chairperson or facilitator in moving the group towards the completion of the task by encouraging collaboration. This, according to Kimball [27] is achieved through a process of relationship building and providing support to the group. The challenge is that the virtual chairperson/facilitator has to manage the tension that is be created when attempting to complete complex work using asynchronous CMC between, on the one hand, the reduced cues effects of narrow bandwidth, and on the other hand, a rich social context. The critical role of the chairperson or facilitator is to cut through this tension and create the conditions necessary for the effective completion of the task.

A significant finding from this study is the recognition of a socio-technical tension operating between the constraints imposed by CMC and the social richness of the virtual group using it. The evidence from this study suggests that even in groups with no history and little virtual group experience, and despite a major de-motivating event (funding being withdrawn from the programme), it is still possible to work effectively and to achieve targets.

Clearly the success of virtual groups is dependent on a range of factors that contribute to a socially rich virtual environment. Our findings suggest that these include five principal influences. These are: the presence of a range of individual participant aspirations and agendas salient to the aims and purpose of the group, participant commitment to and identification with the work of the group, input from a diversity of backgrounds relevant to the work of the group, a website design that enhances and facilitates participation and interaction and finally, the mediating effects of an effective chairperson or group facilitator.

## Discussion

In this paper we have discussed the effects of using asynchronous CMC to support virtual committees in the real world setting of NHS funded research commissioning. These committees were made up of people who had never met before and who had to undertake a complex task within a limited time period and then disband. It is against this background that we have focussed this discussion on how the use of technology affected three factors key to effective group work. These are, building relationships, group cohesion and group commitment [26].

The findings from our research suggest that there was considerable progress in putting these three building blocks of 'groupness' in place during the limited time available. However, a number of challenges imposed by the reduced social cues effects of the narrow bandwidth media being used gave the impression that group development was in some way retarded compared to face-to-face groups.

Set against these challenges there were also a number of difficulties associated with the completion of such complex work as research commissioning using zero-history, time-limited, virtual groups. However, these difficulties, including a major de-motivating event, appeared to have little effect on either the time taken or the effectiveness of the decisions reached. Because of this we focussed on the possibility that a form of socio-technical interaction was taking place and that the dynamic effects of social context were, in a sense, augmenting the technical bandwidth capacity of the media being used.

This, we argue, creates a socio-technical tension. On the one hand there are the constraints imposed by CMC and, on the other, the social richness of the virtual group using it. We suggest that, based on our study, social richness results from a number of elements being present. These include; the motivating effects of personal aspirations and agendas, commitment to and identification with the work of the group, the inclusion of people from a diversity of backgrounds with experience relevant to the work of the group and a website designed to enhance participation and interaction. Of critical importance is that the energy located at the point of socio-technical tension is maximised by the mediation of a skilled and fully prepared chairperson or facilitator.

There are a number of implications that arise from this discussion. First, the careful selection of group members and the importance of reflecting the dimensions (background, experience, personal aspirations, agendas, commitment) that makes up a rich social context. Second, is the significance of the choice of chairperson and their potential to effectively manage a virtual meeting rather than a face-to-face one. Third, is to provide orientation and preparation for all participants that facilitates relationship building and an interactive discipline that is proactive as well as more standard virtual skills. Finally, there is the importance of providing opportunities (using a variety of media) for group building before and during the life of the group.

## Conclusion

Given that it is common for virtual work groups to be formed and then disbanded once a task is completed, more research into how the effectiveness of such groups can be maximised is important. If virtual work groups are to be used by the NHS in the future, then we believe there is a need for well designed 'fine-grained' research into the role of social context and its relationship to the effectiveness of newly formed virtual groups. Equally as important are studies that examine the effects of socio-technical interaction on groups undertaking tasks in the real world of work.

## Competing interests

The author(s) declare that they have no competing interests.

## Authors' contributions

CM conceived the study, and participated in its design and led its coordination.

CM, PM and PY participated equally in the collection and analysis of data.

All authors read and approved the final research report.
